# Ontario Veterinary College

**Published:** 1883-07

**Authors:** 


### Reports of Commencements and Societies.

### ONTARIO VETERINARY COLLEGE.

The examinations were held on March 29th and 30th.

Professor Smith, in opening the proceedings, remarked on the extraordinary success of the College during the past session.

Dr. Duncan then read the names of the graduates, also the Prize List. The prizes were presented by the Lieutenant-Governor and other gentlemen.

### LIST OF GRADUATES.

Henry B. Adair, Paris, Kentucky; James Addison, Newmarket, Ont; Frank H. Armstrong, Ausable, Michigan; Vinton A. Berry, Marion, Ohio; James E. Blackall, Birr, Ont.; Cyrus J. Blank, Coopersburg, Penn.; Elmer E. Bowen, Tyre, New York; Robert W. Carter, Guelph, Ont.; Edward St. Geo. Courtenay, Waterford, Ireland; John B. Crane; Sharen Centre, Ohio; Samuel S. Dickenson, Zion, Ont.; Charles M. Dunn, Hamilton, Ont.; James W. Fisher, Baillieborough, Ont.; Edward R. Forbes, Toronto, Ont.; William R. Howe, Cleveland, Ohio; V. L. James, Springfield New York; Harry F. James, Ottawa, Ont.; George P. Jeffery, Toronto, Ont.; James Johnston, Dundee, Scotland; Robert A. Jones, Simcoe, Ont.; William Jobling, Parkhill, Ont.; Jesse R. Keeler, Harleyville, Pennsylvania; Thomas Kerr, Wingham, Ont.; Charles C. McLean, Meadville, Penn.; George Murray, Ridgetown, Ont.; John Newton, Weston, Ont.; John Perdue, Orangeville, Ont.; Mortimer W. Plank, Uxbridge, Ont.; Marshall M. Poucher, Oswego, N. Y.; Tipton J. Queen, Salineville, Ohio; John F. Quinn, Edmunton, Ont.; William R. Rowe, Bond Eau, Ont.; James W. Sallade, Reading Penn.; Allen S. Shimer, Shimersville, Penn.; Merritt W. Sine, Stirling, Ont.; James F. Smith, Port Ryerse, Ont.; Jacob Stallman, Rochester, N. Y.; John G, Stewart, Brantford, Ont.; Robert W. Stewart, Mt. Victory,

Ohio; George W. Simpson, Mackinaw City, Mich; Albert E. Thompson, Strathroy, Ont.; Joseph B. Thompson, New York; Henry Van Zant, Mongola, Ont.; Jonathan C. Whitney, Allan, Michigan; Willard E. Wight, Millbury, Ohio; J. H. Schoonmaker, New York.

PRIZE LIST—SENIORS.

*Pathology.*—Silver medal, C. C. McLean; second prize, H. F. James; third, H. B. Adair.

*Anatomy.*—Silver medal, H. F. James; second prize, C. C. McLean; third, H. B. Adair, and J. Newton, (equal).

*Entozoa.*—Prize, J. Newton.

*Microscopy.*—Prize, Dunn.

*Chemistry.*—First prize, Jobling; second, Newton; third, Dickenson.

*Physiology.*—First prize, H. F. James; second, Sallade; third, Dunn.

*Anatomical Preparation.*—Silver medal, Adair; second prize, V. L. James.

*Materia Medica.*—First prize, H. F. James; second Sallade; third, Sine.

*Breeding and Management of Stock.*—First prize, Jobling, 20 dols. in books (by Hon. Commissioner of Agriculture); second prize, H. F. James 15 dols. in books, (Council of Agricultural and Arts Association); third prize, Adair, 10 dols. in books (by Agricultural and Arts Association).

Gold medal for best general examination, presented by the Ontario Veterinary Medical Association, H. F. James.

PRIZE LIST—JUNIORS.

*Anatomy.*—Silver medal, L. C. Tiffany; second prize, J. F. Reed; third, G. W. Butler.

*Pathology.*—First prize, J. F. Reed; second, L. C. Tiffany; third, G. W. Butler.

*Chemistry.*—First prize, Silverthorne; second, Ardiel.

*Physiology.*—First prize, H. G. Reed; second, J. H. Reed; third, W. F. Berry.

The Lieutenant-Governor, having been thanked by the President for presenting some of the prizes, and for his presence among them, was requested to make a few observations, when he said to the President and gentlemen that it gave him much pleasure as Lieutenant-Governor of the province to show by his presence there his appreciation of the Ontario Veterinary College. Judging from all that he had at times heard of it, the high status its graduates had attained, both in Canada and in the United States, the increasing number of its students, greater this year than ever before, this College, in his opinion, well deserved any compliment that the Lieutenant-Governor, as a representative man, could by his presence pay to it. He congratulated the fortunate possessors of the respective prizes, but told them that promising and suggestive of success in after-life though these prizes were, they should not run away with the idea that their future advancement was to-day secured. More experience of life would teach them that if they wished to obtain that success which their worthy President had won, they must, like him, have a knowledge of men as well as of animals, cultivate that courtesy, energy, and and judgment in business matters which he had displayed, and which had done so much to place this College and himself in the highly creditable position they now occupied. The veterinary knowledge they were now acquiring was specially useful in Canada, which was the happy and prosperous home of hundreds of thousands of farmers, who, by the increased attention they were now giving to the raising of the best breeds of horses and cattle, were opening up a good field for the profitable employment of those he saw before him. The time was not far distant when the reliable and well-educated veterinary practitioner would take the place in this country of the unreliable and dangerous quack. He wished the College and its students every prospect, some of whom were, no doubt, leaving it for the last time this term, but he hoped they would not soon forget the useful knowledge acquired within its walls. (Applause.)

After remarks from the Mayor, the proceedings terminated.

